# One third of middle ear effusions from children undergoing tympanostomy tube placement had multiple bacterial pathogens

**DOI:** 10.1186/1471-2431-12-87

**Published:** 2012-06-28

**Authors:** Robert C Holder, Daniel J Kirse, Adele K Evans, Timothy R Peters, Katherine A Poehling, W Edward Swords, Sean D Reid

**Affiliations:** 1 Department of Microbiology and Immunology, Wake Forest School of Medicine, Winston Salem, NC, 27101, USA; 2 Department of Otolaryngology-Head and Neck Surgery, Wake Forest School of Medicine, Winston Salem, NC, 27157, USA; 3 Department of Pediatrics, Wake Forest School of Medicine, Winston Salem, NC, 27157, USA; 4 Department of Epidemiology and Prevention, Wake Forest School of Medicine, Winston Salem, NC, 27157, USA

## Abstract

**Background:**

Because previous studies have indicated that otitis media may be a polymicrobial disease, we prospectively analyzed middle ear effusions of children undergoing tympanostomy tube placement with multiplex polymerase chain reaction for four otopathogens.

**Methods:**

Middle ear effusions from 207 children undergoing routine tympanostomy tube placement were collected and were classified by the surgeon as acute otitis media (AOM) for purulent effusions and as otitis media with effusion (OME) for non-purulent effusions. DNA was isolated from these samples and analyzed with multiplex polymerase chain reaction for *Haemophilus influenzae*, *Streptococcus pneumoniae*, *Alloiococcus otitidis*, and *Moraxella catarrhalis*.

**Results:**

119 (57%) of 207 patients were PCR positive for at least one of these four organisms. 36 (30%) of the positive samples indicated the presence of more than one bacterial species. Patient samples were further separated into 2 groups based on clinical presentation at the time of surgery. Samples were categorized as acute otitis media (AOM) if pus was observed behind the tympanic membrane. If no pus was present, samples were categorized as otitis media with effusion (OME). Bacteria were identified in most of the children with AOM (87%) and half the children with OME (51%, p < 0.001). A single bacterial organism was detected in middle ear effusions from children with AOM more often than those with OME (74% versus 33%, p < 0.001). *Haemophilus influenzae* was the predominant single organism and caused 58% of all AOM in this study. *Alloiococcus otitidis* and *Moraxella catarrhalis* were more frequently identified in middle ear effusions than *Streptococcus pneumoniae*.

**Conclusions:**

*Haemophilus influenzae*, *Streptococcus pneumoniae*, *Alloiococcus otitidis*, and *Moraxella catarrhalis* were identified in the middle ear effusions of some patients with otitis media. Overall, we found AOM is predominantly a single organism infection and most commonly from *Haemophilus influenzae*. In contrast, OME infections had a more equal distribution of single organisms, polymicrobial entities, and non-bacterial agents.

## Background

Otitis media is one of the most common childhood diseases [1]. Acute otitis media (AOM) typically exhibits rapid-onset purulent middle ear effusion and symptoms of middle ear inflammation, including fever and otalgia [2], whereas otitis media with effusion (OME) exhibits non-purulent middle ear effusion in the absence of symptoms of acute infection [3]. Otitis media is the leading reason for pediatric office visits and for antibiotic prescriptions [4]. The economic burden of otitis media in the United States is estimated at $3–5 billion in direct annual costs [5,6], and much higher if indirect costs such as lost working days and loss of productivity by family members caring for the sick are included [7].

A common treatment for frequent AOM and for persistent OME with hearing loss is the insertion of tympanostomy tubes. Insertion of tympanostomy tubes is the most common surgical procedure excluding circumcision for U.S. children ≤15 years of age [8,9]. Parents of children who have undergone tympanostomy tube placement report an increased quality of life due to the elimination of AOM symptoms and improved hearing and speech [10].

The bacterial pathogens most commonly cultured from the middle ear effusions of children with AOM are *H. influenzae*, *S. pneumoniae*, and *M. catarrhalis*[11,12]. Although routine bacterial cultures have been the conventional method to identify the etiology of AOM, approximately 35% of these bacterial cultures are negative [13-15]. Polymerase chain reaction (PCR) has detected bacterial DNA in culture-negative middle ear effusions [16,17]. PCR not only provides a more sensitive method for identifying MEE pathogens, but it also allows for the identification of fastidious or slow-growing organisms. For example, *A. otitidis*, a pathogen first isolated from middle ear effusions of children with OME [18], was more recently identified because its slow growth characteristics are not conducive to identification using conventional bacterial culture methods [13]. The sensitivity of PCR has lead to a growing recognition that otitis media may be a polymicrobial disease [19]. Here we used a multiplex PCR to determine the prevalence of four known otopathogens (*H. influenzae*, *S. pneumoniae*, *A. otitidis*, and *M. catarrhalis*) in the middle ear effusions of children undergoing routine tympanostomy placement.

## Methods

### Patients and sample collection

Effusion samples were collected from all children (< 18 y) who had tympanostomy tube placement for clinical indications at Wake Forest School of Medicine from July 2009 through December 2010 provided middle ear fluid was present at the time of surgery. The principle pre-operative diagnoses were recurrent acute otitis media and chronic otitis media with effusion. These are the two principle indications for which children undergo myringotomy and tympanostomy tube placement. The children from whom samples were collected are representative of all children who undergo myringotomy and tympanostomy tube placement at Wake Forest. This study was reviewed and approved by the Wake Forest School of Medicine Institutional Review Board as an exempt study; no personal health information was collected and discarded samples of middle ear effusions were evaluated for bacterial pathogens.

Children who at the time of tympanostomy tube placement had middle ear effusions had that fluid aspirated into a sterile trap. Middle ear effusion samples were categorized as AOM or OME by the surgeon at the time of tympanostomy tube placement. AOM was diagnosed by the identification of purulent fluid behind the tympanic membrane upon myringotomy. OME was defined as non-purulent fluid behind the tympanic membrane. All middle ear effusion samples were kept at room temperature and transported to the research laboratory within 2 h. Samples were then refrigerated at 4°C until DNA isolation. All data was prospectively acquired. While a formal blind was not used, specimen processing was done without prior record review. Once samples were collected, surgeons were not involved in specimen processing.

### Isolation of DNA

For the few effusions that were very viscous, they were placed in Lysing Matrix D tubes (MP Biomedical, Solon, OH) with 500μL of 1X TE buffer (pH 7.5). The lysing tubes were processed in a FastPrep FP120 homogenizer (Thermo Electron Corporation, Milford, MA) for 40 s on a setting of 6.0. Processed samples were centrifuged at 12000 rpm for 5 min and 200μL of supernatant were used as the starting material for genomic DNA extraction.

DNA was isolated from middle ear effusions using a conventional genomic DNA extraction protocol. First, 200μL of effusion were treated with 15μL of Mutanolysin (10U/μL; Sigma, St. Louis, MO) and 21μL of Lysozyme (20 mg/mL; Amresco, Solon, OH) and then incubated at 37°C for 1 h. Second, samples were treated with 55μL of 10% SDS (EMD, Gibbstown, NJ) and 68μL of RNAse A (Sigma, St. Louis, MO) and then incubated at 37°C for 1 h. Third, samples were treated with 10μL of Proteinase K (10 mg/mL; Amresco, Solon, OH) and then incubated at 37°C for 1 h. Fourth, samples were treated with 55μL of 5 M NaCl (Sigma, St. Louis, MO) and 50μL of prewarmed (to 60°C) 10% Hexadecyltrimethyl-ammonium bromide (Sigma, St. Louis, MO) and then incubated at 60°C for 20 min. Fifth, samples were mixed with 470μL of Phenol:Chloroform:Isoamyl Alcohol (25:24:1; Sigma, St. Louis, MO) and transferred to prespun Phase Lock Tubes (2 mL, heavy; 5 Prime, Gaithersburg, MD). Tubes were centrifuged for 5 min at 12000 rpm, and the upper (aqueous) layer from each sample was transferred to a sterile 1.5 mL microcentrifuge tube. Aqueous layers were mixed with 50μL of 3 M NaOAc (Sigma, St. Louis, MO) and 470μL of ice cold 100% Ethanol (Warner-Graham, Cockeysville, MD). Samples were incubated at −20°C for a minimum of 30 min. Precipitated samples were centrifuged for 30 min at 12000 rpm at 4°C. Supernatants were discarded and pellets washed with 1 mL of 70% Ethanol. Pellets were allowed to completely dry and were resuspended in a final volume of 100μL of dH_2_O.

### Polymerase chain reaction

**Figure 1:**
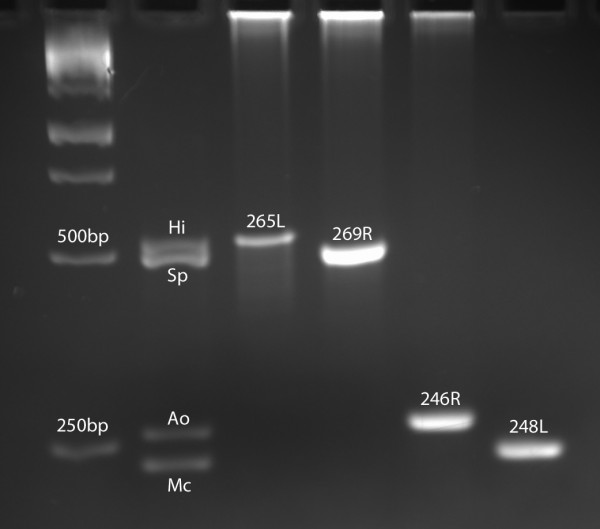
**Multiplex PCR Results. Multiplex PCR products were electrophoresed on 2.5% agarose.** Hi, *Haemophilus influenzae* control band, expected size 525 bp; Sp, *Streptococcus pneumoniae* control band, expected size 484 bp; Ao, *Alloiococcus otitidis* control band, expected size 264 bp; Mc, *Moraxella catarrhalis* control band, expected size 237 bp; 265L, 269R, 246R, and 248L, experimental samples; bp, base pair.

A multiplex PCR procedure was performed to simultaneously detect *H. influenzae*, *S. pneumoniae*, *A. otitidis*, and *M. catarrhalis* with minor modifications of the methods described by Hendolin et al. [20]. Briefly, PCR extension steps were performed at 65°C with HotMasterMix (5 Prime, Gaithersburg, MD) and using the primers listed in Table 1. *H. influenzae* 86–028, *S. pneumoniae* TIGR4, *A. otitidis* SS1337, and *M. catarrhalis* 7169 were used as positive controls for PCR (Figure 1). Results were visualized using agarose gel electrophoresis (2.5% agarose, 100 V for 3 h) and ethidium bromide staining (500 ng/mL final ethidium bromide concentration).

**Table 1:** Primers used in multiplex PCR

** Primer Name**	**Nucleotide Sequence**	**Predicted Amplicon Size^a^**
*H. influenzae* FWD	5′-CGTATTATCGGAAGATGAAAGTGC-3′	525 base pairs
*S. pneumoniae* FWD	5′-AAGGTGCACTTGCATCACTACC-3′	484 base pairs
*A. otitidis* FWD	5′-GGGGAAGAACACGGATAGGA-3′	264 base pairs
*M. catarrhalis* FWD	5′-CCCATAAGCCCTGACGTTAC-3′	237 base pairs
Universal REV	5′-CTACGCATTTCACCGCTACAC-3′	

^a^When the designated FWD primer is used in conjunction with the Universal REV primer.

### Chart review

The demographic characteristics, current medications, and past medical history were all determined by chart review performed at the time of surgery without collecting any personal health identifiers. The pediatric otolaryngologist used a standardized clinical form that elicited all of this clinical information by history and/or by documentation from the primary care provider. Previous antibiotics were documentation of any course of antibiotics within 6 months of surgery.

### Analyses

The frequency of demographic characteristics, clinical characteristics, or microbiologic results of children with AOM and OME middle ear effusions were compared using chi-square analyses or Fisher’s exact tests. For the children who had middle ear effusions in both ears, the results from each ear were combined so that all children with one or two middle ear effusions had one result. Identification of purulence in the effusion of at least one ear was used as a basis for categorizing the sample as AOM. STATA 8.1 was used for all statistical analyses.

## Results

The study population comprised 207 children undergoing tympanostomy tube placement for clinical indications at Wake Forest School of Medicine from July 2009 to December 2010. Two-thirds of the study population was male, half were 1–3 years of age, and 60% were Caucasian (Table 2). Children with AOM at tympanostomy were more likely to be younger, to have had previous ear infections, or to have been treated with antibiotics in the previous 6 months than children with OME at tympanostomy. In contrast, children with OME were more likely to have had an adenoidectomy than children with AOM.

**Table 2:** Child Demographics

	**Total**	**Purulent Effusions**	**Nonpurulent Effusions**	
	No. of Children (column %) (n = 207)	No. of Children (column %) (n = 38)	No. of Children (column %) (n = 169)	p-value
Age				
<1	33 (16)	5 (13)	28 (17)	0.03
1–3	103 (50)	25 (66)	78 (46)	
>3	69 (33)	7 (18)	62 (37)	
Gender				
Male	139 (67)	21 (55)	118 (70)	0.09
Female	68 (33)	17 (45)	51 (30)	
Race				
White	123 (60)	29 (76)	94 (56)	0.09
Black	44 (21)	5 (13)	39 (23)	
Hispanic	36 (17)	3 (8)	33 (20)	
Other	2 (1)	0 (0)	2 (1)	
Currently on Antibiotics^a^				
Yes	26 (13)	7 (18)	19 (11)	0.27
No	179 (86)	30 (79)	149 (88)	
Currently on Allergy Medicines^b^				
Yes	44 (21)	8 (21)	36 (21)	1.00
No	160 (77)	30 (79)	130 (77)	
Previous Ear Infections				
Yes	145 (70)	33 (87)	112 (66)	0.008
No	57 (28)	4 (11)	53 (31)	
Previously on Antibiotics^c^				
Yes	100 (48)	26 (68)	74 (44)	0.007
No	107 (52)	12 (32)	95 (56)	
Adenoidectomy				
Yes	16 (8)	0 (0)	16 (9)	0.046
No	186 (90)	38 (100)	148 (88)	
Previous Ear Tubes				
Yes	49 (24)	6 (16)	43 (25)	0.21
No	153 (74)	32 (84)	121 (72)	
Cleft Palate				
Yes	9 (4)	1 (3)	8 (5)	1.00
No	189 (91)	37 (97)	152 (90)	

^a^Current antibiotics include: Amoxicillin, Amoxicillin + Clavulanic Acid, Azithromycin, Cefdinir, Cefpodoxime, Cefprozil, Sulfamethoxazole.

^b^Current allergy medicines include: Budesonide, Cetirizine, Clemastine, Diphenhydramine, Fexofenadine, Fluticasone, Levocetirizine, Loratadine, Mometasone, Montelukast, Olopatadine.

^c^Previous antibiotics include: Amoxicillin, Amoxicillin + Clavulanic Acid, Azithromycin, Cefdinir, Cefpodoxime, Cefprozil, Ceftriaxone, Cefuroxime, Ciprofloxacin, Clindamycin, Sulfamethoxazole.

**Figure 2:**
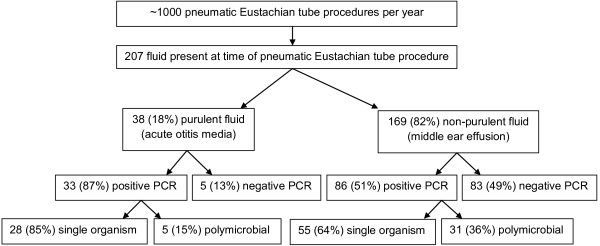
Flow Chart of PCR Analysis.

Effusions from 119 (57%) of 207 children were PCR positive for *H. influenzae, S. pneumoniae, A. otitidis,* and/or *M. catarrhalis*. Of these 119 PCR positive samples, 36 (30%) had 2–4 bacterial species detected (Figure 2, Table 3, Additional file 1: Table S1). Bacteria were identified in 33 (87%) of 38 children with AOM as compared to 86 (51%) of 169 children with OME (p < 0.001). Single bacterial species were identified in the majority of children with AOM and a minority of children with OME (74% versus 33%, p < 0.001). Identifying multiple bacterial pathogens was similar for children with AOM and OME (13% versus 18%, p = 0.64), whereas not identifying any of the four bacterial pathogens was less common for AOM than OME (13% versus 49%, p < 0.001).

**Table 3:** Incidence of bacterial DNA presence in Middle Ear Effusions

	**Purulent Effusions**	**Nonpurulent Effusions**	
Presence of Bacterial DNA	No. of Children (column %) (n = 38)	No. of Children (column %) (n = 169)	p-value
*Haemophilus influenzae*			
Single organism	22 (58)	20 (12)	<0.001
Polymicrobial component	3 (8)	20 (12)	
None	13 (34)	129 (76)	
*Streptococcus pneumoniae*			
Single organism	1 (3)	2 (1)	0.79
Polymicrobial component	1 (3)	6 (4)	
None	36 (95)	161 (95)	
*Alloiococcus otitidis*			
Single organism	2 (5)	22 (13)	0.40
Polymicrobial component	5 (13)	21 (12)	
None	31 (82)	126 (75)	
*Moraxella catarrhalis*			
Single organism	3 (8)	11 (7)	0.95
Polymicrobial component	4 (11)	18 (11)	
None	31 (82)	140 (83)	
Overall			
Single organism	28 (74)	55 (33)	<0.001
Polymicrobial	5 (13)	31 (18)	
No Bacteria Detected	5 (13)	83 (49)	

*Percent totals may not add up to 100% due to rounding error.

For middle ear effusions with a single bacterial species identified, the etiology for children with AOM differed significantly from those with OME (p < 0.001). *H. influenzae* accounted for 79% of the isolates from children with AOM, whereas three bacteria accounted for the majority of isolates for children with OME: *H .influenzae* for 36%, *A. otitidis* for 40% and *M. catarrhalis* for 20%. *S. pneumoniae* was detected in 5% of all isolates for children with AOM and OME. *Alloiococcus otitidis* was identified either as a single organism or as a polymicrobial component in 18% of AOM and 25% of OME. All four bacteria were identified in at least one of the middle ear effusions with AOM or OME.

## Discussion

Using a multiplex PCR to simultaneously detect four bacterial otopathogens in the middle ear effusions obtained from children undergoing routine tympanostomy tube placement, we found distinct bacterial profiles for AOM and OME. Bacteria were identified in most of the children with AOM (87%) and half the children with OME (51%, p < 0.001). A single bacterial organism was detected in middle ear effusions from patients with AOM more often than those with OME (74% versus 33%, p < 0.001). *Haemophilus influenzae* was the predominant single organism and caused 58% of all AOM in this study. *Alloiococcus otitidis* and *Moraxella catarrhalis* were more frequently identified in middle ear effusions than *Streptococcus pneumoniae*.

Overall, we found AOM is predominantly a single organism infection, whereas, OME infections had a more equal distribution of single organisms, polymicrobial entities, and non-bacterial agents.

*H. influenzae* was the predominant bacterial species identified, comprising 66% (25 of 38) of middle ear effusions with AOM and 24% (40 of 169) of middle ear effusions with OME. We observed a higher proportion of samples with *H. influenzae* and a lower proportion with *S. pneumoniae* than what has been previously reported. Kaur et al., using a similar multiplex PCR approach on AOM middle ear effusion, detected *H. influenzae* in 31% of children who were on antibiotic prior to sample acquisition and 39% in children who had not undergone antibiotic treatment [13]. However, Kaur et al. utilized specifically culture-negative MEF, a distinction we have not made with the samples obtained for our research. In other work using culture-negative AOM MEE samples, Xu et al. observed an *H. influenzae* percentage of 24% [21].

While the levels of *H. influenzae* in our patients appear to be higher than what others have seen, we observed the opposing trend with respect to *S. pneumoniae*. *S. pneumoniae*, regarded as one of the 3 most prevalent bacterial contributors to OM infection, was identified in only 2 of 38 AOM patients (~5%) and 8 of 169 OME patients (~5%). In the studies listed above by Kaur et al. and Xu et al., *S. pneumoniae* was identified in around 38%–57% of culture-negative AOM MEF samples, percentages that are considerably higher than what was observed in our study [13,21].

A number of factors may explain our finding that *S. pneumoniae* was detected in only 5% of all study subjects. Published studies of pneumococcal prevalence in otitis media report a wide incidence range. Brooke et al. used standard culture based techniques to detect *S. pneumoniae* in approximately 16% of OME effusions [22]. Post et al. used PCR to detect *S. pneumoniae* in OME effusions at a higher percentage of approximately 30% [23]. Research conducted by Hendolin et al. detected *S. pneumoniae* in only 8% of cases examined [20]. We used the same multiplex PCR primers used by Hendolin et al. Kaur et al. used these PCR primers to identify *S. pneumoniae* in approximately 57% of effusions from children with AOM [13], and this data does not suggest that our PCR assay has a low sensitivity for detection of pneumococcus.

The low *S. pneumoniae* incidence found in our study might be explained by the effectiveness of pneumococcal conjugate vaccination. A heptavalent pneumococcal conjugate vaccine (PCV7) was introduced in 2000, and since its acceptance for widespread use there has been a shift in the incidence of otitis media pathogens. In the years immediately following PCV7 introduction, *H. influenzae* emerged as the most common AOM isolate [14,24]. More recently, *S. pneumoniae* serotypes not included in the PCV7 vaccine have been increasingly isolated from AOM cases [12]. In 2010, a new modified pneumococcal conjugate vaccine was introduced to combat the emergence of *S. pneumoniae* serotypes not included in the original PCV7 vaccine. It contained components from 13 serotypes of *S. pneumoniae* (7 serotypes included in the PCV7 vaccine along with 6 recently emerging serotypes). Our findings are consistent with improved pneumococcal vaccine prevention in children enrolled in this study.

Another interesting result was the high incidence of *A. otitidis* in our OME samples (approximately 25%, single and co-infections included). *A. otitidis* was first identified in OME samples by Faden et al. [18]. It is a Gram-positive organism that exhibits very slow growth, making it very difficult to identify through standard culture techniques. It has been thought of as solely an OME pathogen; however, it is increasingly recognized as a pathogen in AOM [13,25,26]. Our findings support what has been reported previously in OME cases [20]. The *A. otitidis* results further demonstrate the importance of using methods other than standard culture for the identification of fastidious otopathogens.

## Conclusions

In conclusion, the etiology of OM appears to revolve around disease type (AOM or OME). A multiplex PCR approach may be used to identify specific bacterial DNA species in effusions from children experiencing OM. The PCR procedures can overcome the obstacles of culturing fastidious organisms, and may offer a more sensitive and time efficient method for evaluating middle ear effusions. While our approach targeted 4 organisms, the method could be adapted for the identification of additional microorganisms.

Our criterion for separating AOM cases from OME cases was the presence of pus behind the tympanic membrane at the time of tympanostomy tube placement. The results of our research clearly show that this single easily observable patient difference was sufficient to categorize disease condition into 2 distinct populations. Moreover, our results indicate that when AOM is observed there is usually a single bacterial etiology. Culture or PCR analysis of pus at tympanostomy tube placement may be especially useful in guiding antibiotic therapy.

## Competing interests

The authors declare that they have no competing interests.

## Author’s contributions

RCH participated in the design of the study, experimental procedures, data analysis, and manuscript preparation. DJK and AKE participated in the design of the study, sample collection surgeries, data analysis, and manuscript preparation. TRP, KAP, and WES participated in the design of the study, data analysis, and manuscript preparation. SDR participated in the design of the study, experimental procedures, data analysis, and manuscript preparation. All authors gave final approval of the completed manuscript.

## Pre-publication history

The pre-publication history for this paper can be accessed here:

http://www.biomedcentral.com/1471-2431/12/87/prepub

## Supplementary Material

Additional file 1 **Supplemental Table 1:** Incidence of Bacterial DNA Presence in Middle Ear Effusions. Click here for file
