# Supplementary material for: One third of middle ear effusions from children undergoing tympanostomy tube placement had multiple bacterial pathogens
Source: BMC Pediatr. 2012 Jun 28;12:87. doi: 10.1186/1471-2431-12-87 (PMC3475091; doi:10.1186/1471-2431-12-87)
Supplement: Additional file 1 — Supplemental Table 1: Incidence of Bacterial DNA Presence in Middle Ear Effusions. [file 1471-2431-12-87-S1.doc]

| Supplemental Table 1.  Incidence of Bacterial DNA Presence in Middle Ear Effusions | | | | | | | |
| --- | --- | --- | --- | --- | --- | --- | --- |
|  | | Purulent Effusions | | | Nonpurulent Effusions | | |
| Organism Identified |  | No. of Patients  (n = 38) | Percentage^a^ | 95% Confidence  Intervals | No. of Patients  (n = 169) | Percentage^b^ | 95% Confidence  Intervals |
| *Haemophilus influenzae* |  | 22 | 58 | 41-74 | 20 | 12 | 8-18 |
| *Streptococcus pneumoniae* |  | 1 | 3 | 0.07-15 | 2 | 1 | 0.01-3 |
| *Alloiococcus otitidis* |  | 2 | 5 | 1-19 | 22 | 13 | 8-19 |
| *Moraxella catarrhalis* |  | 3 | 8 | 1-19 | 11 | 7 | 4-12 |
| *H. influenzae*  *S. pneumoniae* |  | 0 | 0 |  | 2 | 1 | 0.4-5 |
| *H. influenzae*  *A. otitidis* |  | 1 | 3 | 0.07-15 | 8 | 5 | 2-9 |
| *H. influenzae*  *M. catarrhalis* |  | 0 | 0 | 0.07-15 | 7 | 4 | 2-8 |
| *S. pneumoniae*  *A. otitidis* |  | 0 | 0 |  | 1 | 1 | 0.01-3 |
| *S. pneumoniae*  *M. catarrhalis* |  | 0 | 0 |  | 1 | 1 | 0.01-3 |
| *A. otitidis*  *M. catarrhalis* |  | 2 | 5 | 1-19 | 9 | 5 | 2-9 |
| *H. influenzae*  *S. pneumoniae*  *A. otitidis* |  | 0 | 0 |  | 2 | 1 | 0.1-4 |
| *H. influenzae*  *S. pneumoniae*  *M. catarrhalis* |  | 0 | 0 |  | 0 | 0 |  |
| *H. influenzae*  *A. otitidis*  *M. catarrhalis* |  | 1 | 3 | 0.07-15 | 1 | 1 | 0.01-3 |
| *S. pneumoniae*  *A. otitidis*  *M. catarrhalis* |  | 0 | 0 |  | 0 | 0 |  |
| *H. influenzae*  *S. pneumoniae*  *A. otitidis*  *M. catarrhalis* |  | 1 | 3 | 0.07-15 | 0 | 0 |  |
| None detected |  | 5 | 13 | 3-26 | 83 | 49 | 41-57 |

^a^Percentage of purulent effusion isolates only (rounded to nearest whole number)

^b^Percentage of nonpurulent effusion isolates only (rounded to nearest whole number)
